# Supplementary material for: Genome Resequencing for Autotetraploid Rice and Its Closest Relatives Reveals Abundant Variation and High Potential in Rice Breeding
Source: Int J Mol Sci. 2024 Aug 19;25(16):9012. doi: 10.3390/ijms25169012 (PMC11354466; doi:10.3390/ijms25169012)
Supplement: Supplementary file 1 [file ijms-25-09012-s001.zip › 3-1 Supplementary Figures 2023-10-30.pdf]

Supplementary Figures

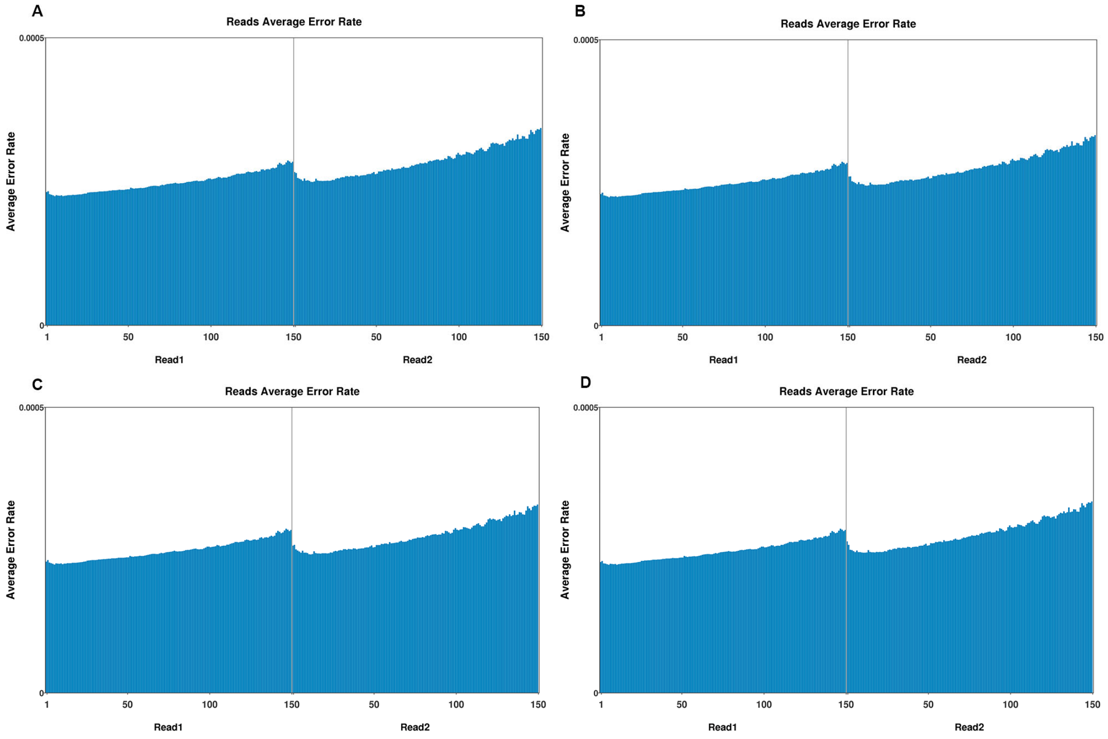

Figure S1. The distribution of base error rate of reads in double-end sequencing. A, Base error rate distribution of sample HT2; B, Base error rate distribution of sample HT4; C, Base error rate distribution of sample HTRM2; D, Base error rate distribution of sample HTRM12. The horizontal coordinates indicate the base positions on reads, and the vertical coordinates indicate the average error rate of single bases. The average base error rate distribution of R1-end reads in double-end sequencing is shown on the left, and the average base error rate distribution of R2-end reads is shown on the right.

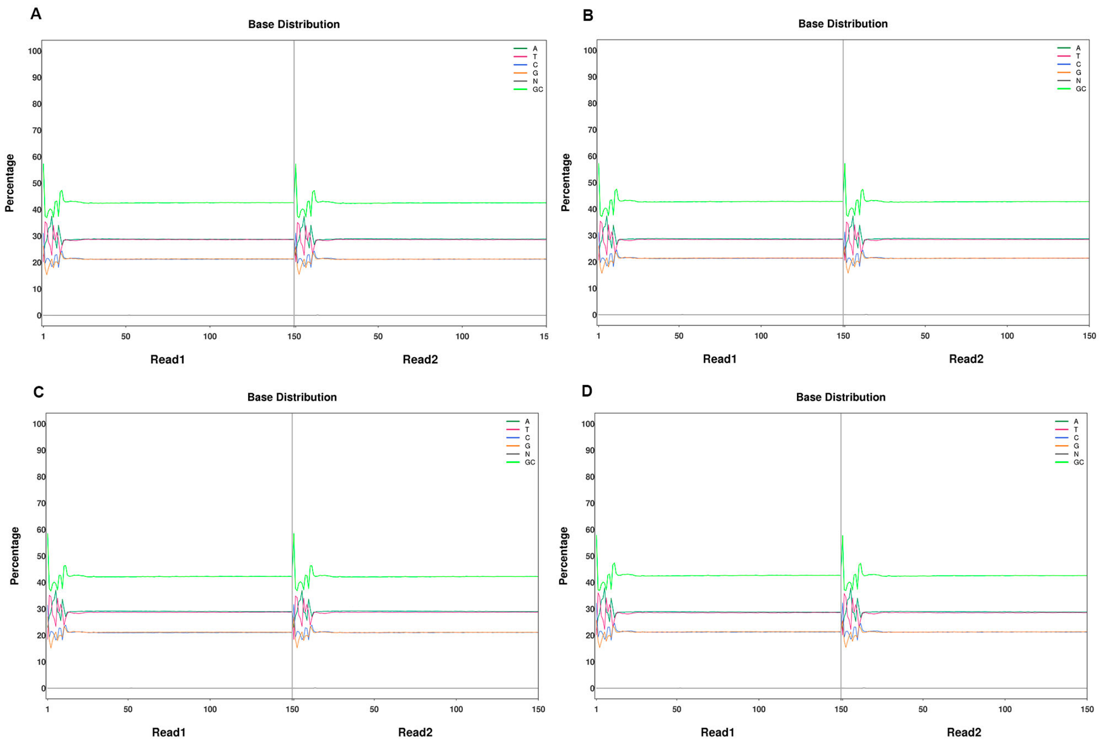

Figure S2. The proportion distribution of base of reads in double-end sequencing. A, Base percentage distribution of HT2; B, Base percentage distribution of HT4; C, Base percentage distribution of HTRM2; D, Base percentage distribution of HTRM12; The horizontal coordinate indicates the position of bases on reads, and the vertical coordinate indicates the proportion of single bases. A, T, C, G, N, and GC indicate the proportion of six types of bases, A, T, C, G, N, and G+C, respectively.

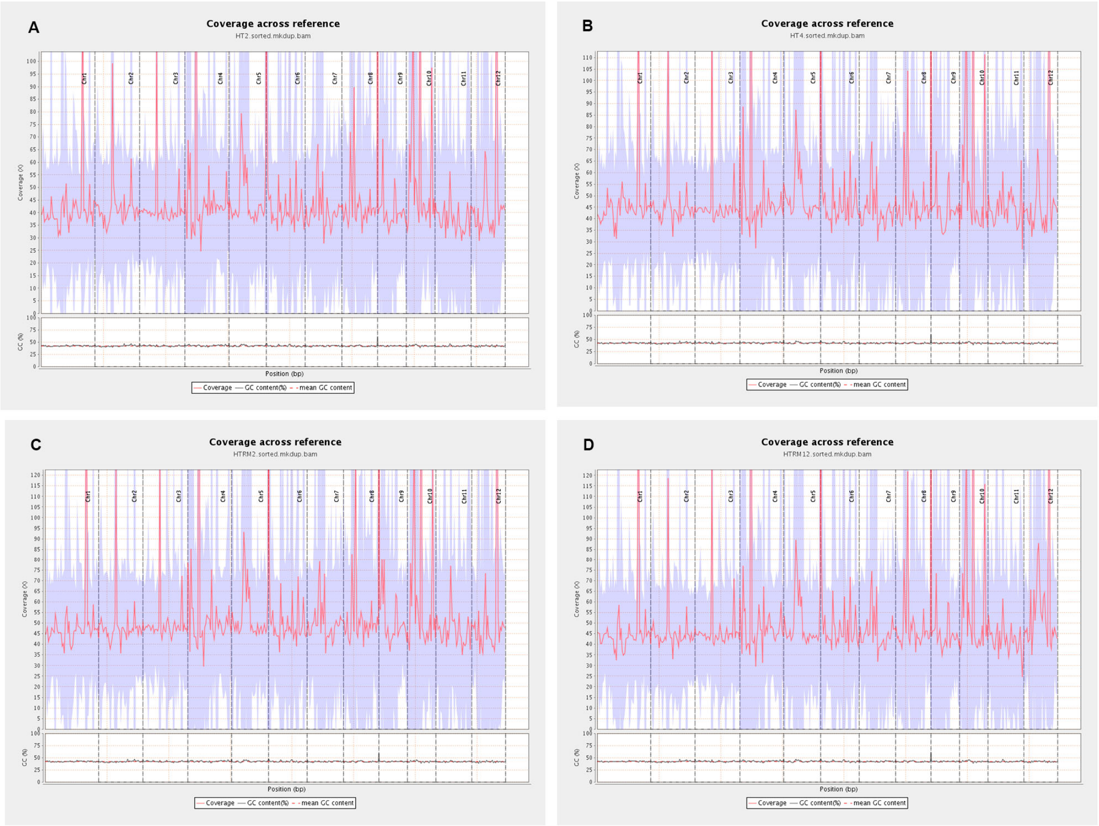

Figure S3. Genome coverage distribution. A, Genome-wide coverage distribution of HT2; B, Genome-wide coverage distribution of HT4; C, Genome-wide coverage distribution of HTRM2; D, Genome-wide coverage distribution of HTRM12. The horizontal coordinate indicates the physical location of the chromosome and the vertical coordinate indicates the average number of covered reads.

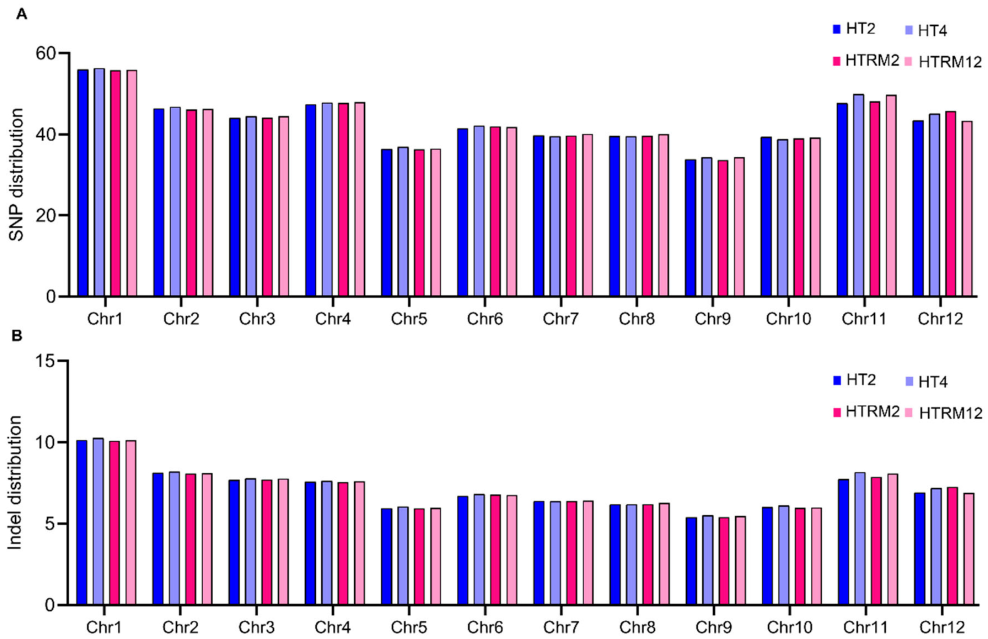

Figure S4. Distribution of single nucleotide polymorphisms (SNPs) and insertion-deletions (InDels) on different chromosomes for each sample. A, The distribution of SNPs on different chromosomes for each sample; B, The distribution of Indels on different chromosomes for each sample.

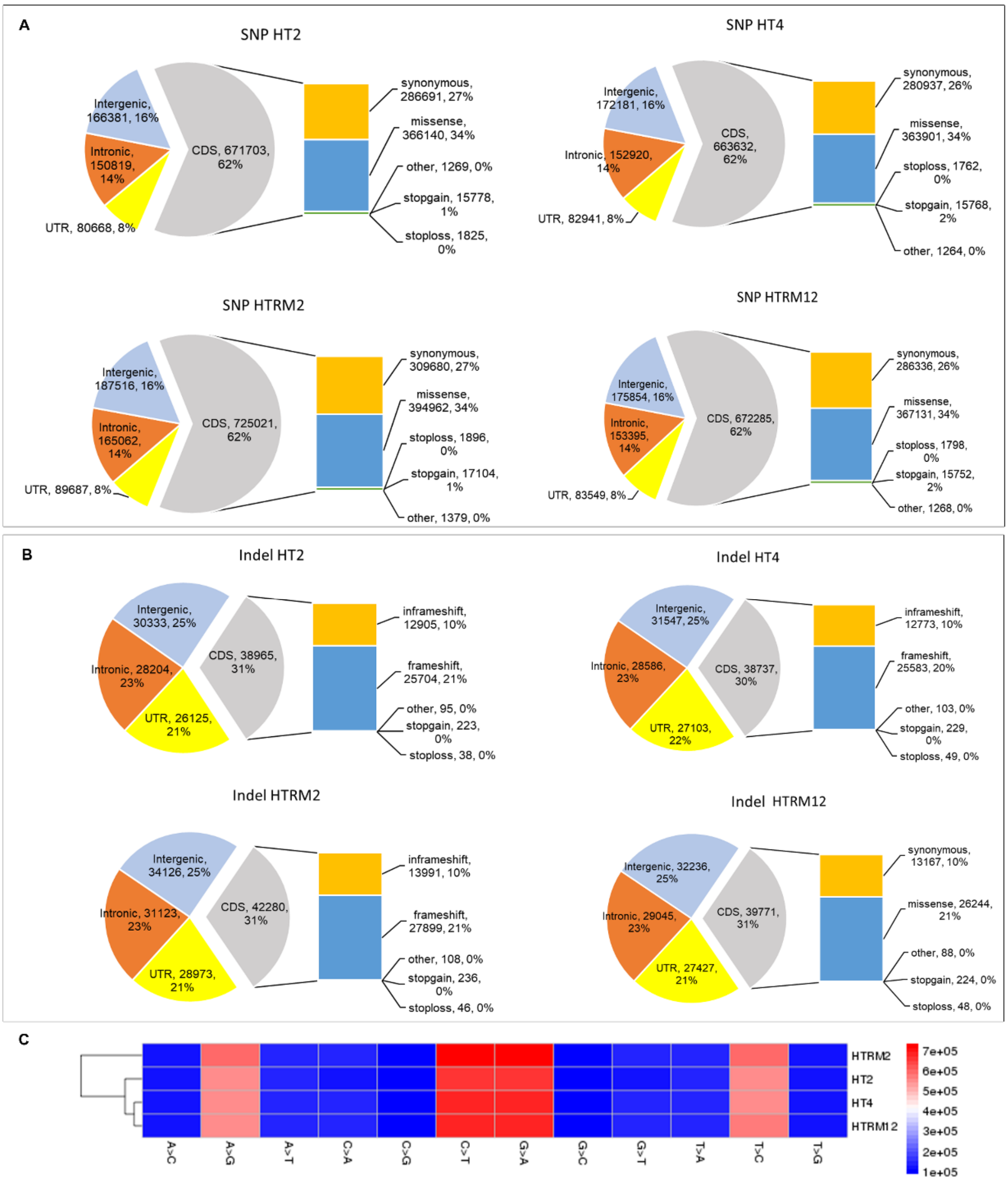

Figure S5. Single nucleotide polymorphism (SNP) and insertion-deletion (InDel) annotations as well as SNP mutation type statistics for each sample. A, SNP annotations for each sample; B, InDel annotations for each sample; C, Heat map of SNP substitution type spectrum by sample. The horizontal coordinate indicates the type of SNP mutation, the vertical coordinate is each sample, and the color shade indicates the number of SNPs.

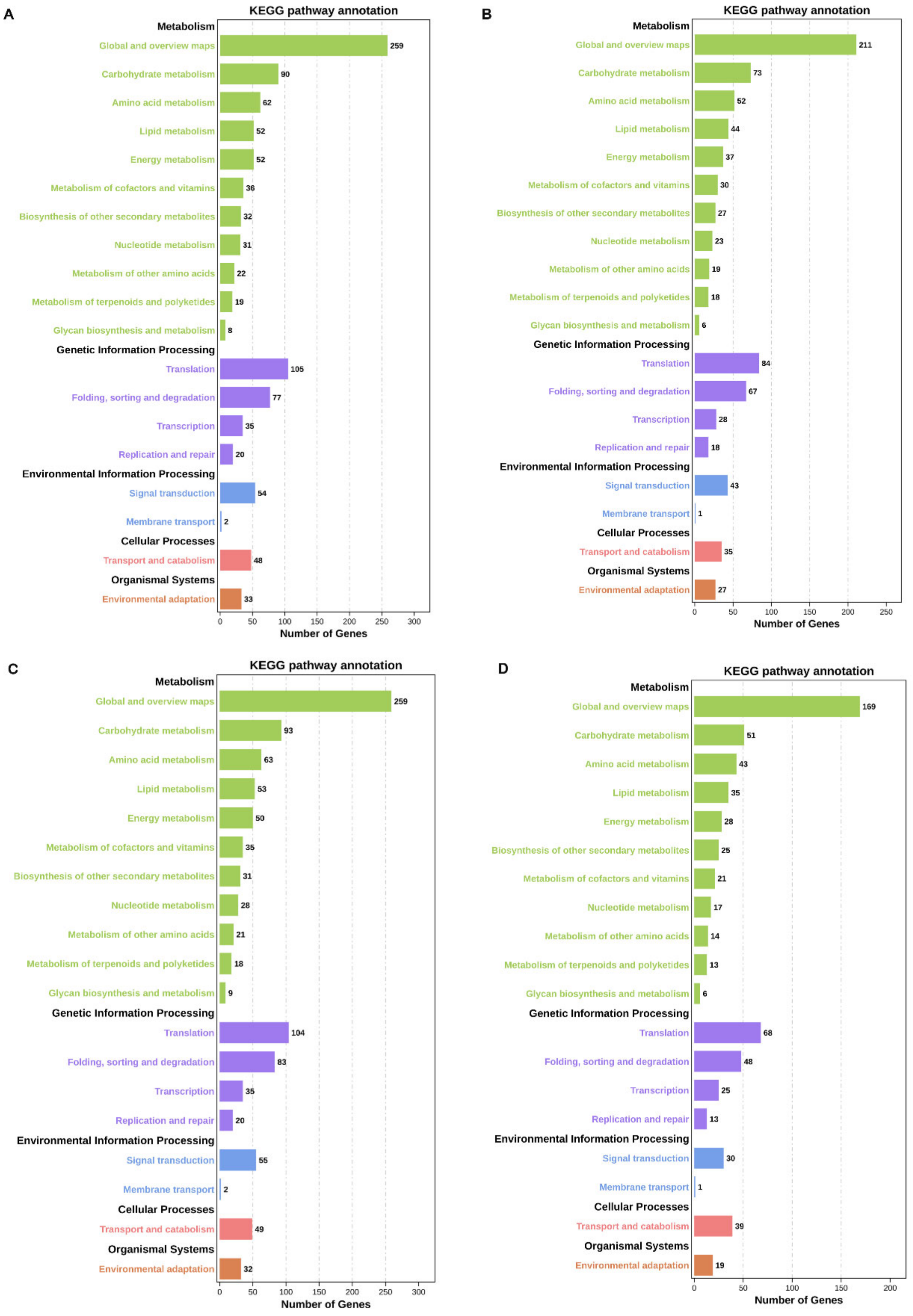

Figure S6. Kyoto Encyclopedia of Genes and Genomes (KEGG) enrichment analysis of genes with homozygous insertion-deletion (InDel) mutations in each sample. A, KEGG enrichment analysis of genes with homozygous InDel mutation in HT2; B, KEGG enrichment analysis of genes with homozygous InDel mutation in HT4; C,

KEGG enrichment analysis of genes with homozygous InDel mutation in HTRM2; D, KEGG enrichment analysis of genes with homozygous InDel mutation in HTRM12. The abscissa represents the number of genes and the ordinate represents various KEGG pathways. The different colors represent different KEGG pathways.
